# The Medicolegal Implications of Functional Neurological Disorder

**DOI:** 10.1055/a-2820-4053

**Published:** 2026-03-24

**Authors:** Mahinda Yogarajah, Tiago Teodoro, Niruj Agrawal

**Affiliations:** 1 Department of Epilepsy, UCL Queen Square Institute of Neurology, London, United Kingdom; 2 Chalfont Centre for Epilepsy, St Peter, United Kingdom; 3 Biomedical Research Centre, NIHR University College London Hospitals, London, United Kingdom; 4 Atkinson Morley Regional Neuroscience Centre, St Georges University Hospital, London, United Kingdom; 5 Department of Neuropsychiatry, South West London and St George's Mental Health Trust, London, United Kingdom

**Keywords:** functional neurological disorder, medicolegal, malingering, symptom validity testing, factitious disorder

## Abstract

Functional neurological disorder (FND) commonly arises in the context of injury, trauma, or litigation and poses distinctive medicolegal challenges. This review outlines how positive clinical signs and contemporary neurobiological models support FND as a robust diagnosis, while distinguishing it from factitious disorder and malingering. We discuss approaches to assessing authenticity of reported disability, the role and limitations of symptom and performance validity tests, and the pitfalls of equating inconsistency with deception. We then consider causation within common legal frameworks using a predisposing–precipitating–perpetuating model to analyze the contribution of index events, background vulnerabilities, and the litigation process itself. Finally, we summarize current evidence on treatment and prognosis, highlighting the limits of prediction and the need to offer courts transparent, probabilistic opinions on long-term outcome and care needs. Our aim is to support clinicians in providing balanced, clinically grounded expert evidence for patients with FND.

## Introduction

Functional neurological disorder (FND) presents a unique set of medicolegal challenges due to its complex clinical presentation, perceived diagnostic ambiguity, and the intersection of neurology, psychiatry, and the law. FND often arises after physical trauma or psychological stress, making it a frequent complication in personal injury and compensation claims, and a subject of forensic assessment in both civil and criminal legal contexts. Given the differences in legal systems between countries the purpose of this article is to focus on the general medicolegal issues that arise in individuals diagnosed with FND as opposed to specific legal aspects such as driving which may differ internationally. The core medicolegal issues include the issue of misdiagnosis, difficulties in differentiating FND from malingering or factitious disorders, and questions around causation and prognostication ( Table 1 ). The reader is referred to the other articles in this special issue that cover in detail the definition, diagnosis, mechanisms, management, and prognosis of FND. We will restrict our comments on these topics to areas where there are medicolegal implications of FND.

**Table 1:** Core medicolegal issues arising in FND cases

Theme	Core medicolegal questions
Diagnosis and misdiagnosis	Is the diagnosis secure? Could this be an alternative neurological disorder?
Authenticity of symptoms	Are symptoms involuntary (genuine) versus intentionally produced/exaggerated?
Causation	Did the index event materially contribute to onset/worsening of FND?
Prognosis	What is likely trajectory and level/duration of impairment?
Treatment and rehabilitation	What treatment is reasonable, necessary, and proportionate?
Ongoing care, aids and litigation	What ongoing support is justified, and how may litigation context affect recovery?

Abbreviation: FND, functional neurological disorder.

## Diagnosis and Misdiagnosis

FND is one of the most common forms of neurological disorder. 1 It is related to malfunctioning of the brain but not (directly) caused by structural damage to the nervous system. FND may involve multiple domains of the nervous system, including motor control, sensory processing, cognition, and/or consciousness. Accordingly, its manifestations can be very diverse, encompassing seizures, weakness, tremor, numbness, subjective cognitive difficulties, and/or altered awareness/responsiveness (dissociation).

FND can be diagnosed using the diagnostic criteria set out by the *Diagnostic and Statistical Manual* , Fifth Edition, Text Revision (DSM V) 2 or the International Classification of Diseases, 11 ^th^ Revision (ICD 11). 3 In essence the diagnosis is based upon signs of inconsistency and incongruency. 4 5 The former refers to inconsistency between movement, sensory or cognitive performance in a voluntary versus an “automatic” scenario. Many positive clinical signs (e.g., gradual onset symptoms, Hoover's sign of functional leg weakness, the tremor entrainment test for functional tremor, and a range of cognitive tests in functional cognitive disorder) used to support a diagnosis of FND are based on this principle. Incongruency, on the other hand, refers to a clinical feature that is not present in other superficially similar neurological conditions, or features that violate laws of anatomy, biology, or physics (e.g., the presence of a tubular visual field deficit where the size of the visual field remains the same regardless of the distance from the object, which is inconsistent with the laws of optics and eye physiology).

By using positive features to diagnose FND, avoiding cognitive traps (e.g., placing too much emphasis of the psychosocial history, “la belle indifference,” normal imaging, or a specific patient “type”), looking for common comorbidities (e.g., fatigue, pain, or cognitive symptoms such as subjective memory or word finding complaints) and being alert to unusual “organic” neurological disorders (e.g., frontal lobe seizures, stiff person syndrome, dystonia), it is possible to make a clinical diagnosis of FND with significant robustness. 6 Indeed, it is the experience of the authors of this article that many patients referred for a specific condition or symptoms in the context of medicolegal cases often have undiagnosed FND. This is in keeping with the literature which suggests that misdiagnosis rates of FND are low at approximately 4% and that rates of a missed diagnosis of FND are far higher. 7 8 9

## The Authenticity of Symptoms—Involuntary versus Deliberate Exaggeration/Falsification

Once the diagnosis of FND has been established in a medicolegal case, one of the key issues in many cases is the extent to which an individual's symptoms are thought to be the product of conscious choice, or a neuropsychiatric disorder (i.e., FND for the purpose of this article) and beyond the individual's volition or intention, and/or perhaps both. While in clinical practice there is significant and multifaceted evidence that patients with FND are not feigning their symptoms, 10 in a medicolegal context it is acknowledged that deliberate exaggeration or falsification of symptoms may be more common. Ultimately this is a decision for the court to answer, but the clinically informed professional view of a medicolegal expert also has a role to play, given that a routine part of his or her clinical practice and experience is assessing the veracity of reported symptoms. It is therefore important to address the question of how much of the disability in a patient with FND is exaggerated or intentional.

Feigned symptoms are encountered when a patient intentionally gives a false account of symptoms or pretends to have impaired function. Classically symptoms produced volitionally have been classified into two categories. 11 Patients with factitious disorder deliberately feign/produce symptoms and deceive clinicians for the purpose of becoming a patient and obtaining medical care. Their motivation is ascribed to an often (but not always) sub- or pre-conscious internal or psychological drive to assume the sick role, which in theory leads to emotional gratification from attention and care. Malingering is the intentional faking or exaggeration of symptoms for an obvious consciously aware external benefit, such as financial compensation, housing, medications, avoiding work, or criminal prosecution. Malingering is considered a behavior and not a psychiatric disorder. In practice however, this categorical approach is limited, and many advocate that it is better to conceptualize these types of behavior in a dimensional manner. Two axes have been proposed; an axis of intentional symptom production (ranging from no intentional symptom production such as in FND to high levels of intentional symptom production such as in malingering or factitious disorder) and another axis of motivation and awareness (ranging from internal and possibly sub- or pre-conscious motivation in factious disorder to external, conscious motivation in malingering). 12 Conversely, there is a substantial body of evidence, including behavioral, neuroimaging, and neurophysiological studies, suggesting that FND, factitious disorder, and malingering are underpinned by different mechanisms, rather than being part of a common continuum of disorders. 10

Given that in many medicolegal cases the level of compensation is dependent on the level of disability reported, the fundamental question is whether that level of reported disability is accurate. For this reason, therefore, it is the first axis, or degree of intentional symptom production, that is critical. Questions of motivation are not relevant to the determination of levels of compensation, and in many respects one's opinion on motivation is likely to simply reflect personal biases. However, there are no tests that can confidently identify symptoms which are intentionally produced. Therefore, the first step in addressing this question is to understand in detail the nature of the impairments reported by the patient, and the kinds of specific disability resulting from them in day-to-day life. The second step is to then assess the level of objective disability and function. The third step is to assess the discrepancy between claimed function and actual intentional function. Here, intentional function means situations where patients are likely to have explicit conscious awareness of their functioning. This is an important distinction because by definition body awareness is altered in FND. Indeed, one of the fundamental mechanisms relevant to the development of FND is attentional dysfunctional (bodily hyper- or hypo-vigilance). For example, Hoover's sign, which is a classical neurological sign of functional leg weakness, is based on the principle that leg weakness improves pre-consciously when attention is diverted away from the leg and worsens when attention is directed toward the leg. Minor discrepancies in symptoms are therefore in keeping with the variable nature of FND symptoms where attention can pre-consciously modulate symptoms, and which tend to worsen when attention is paid to them. For the same reason people with FND often struggle to give accurate accounts of how severe their symptoms are and how long they last. In one study of individuals with either functional or organic tremor, participants wore devices that continuously measured tremor and completed tremor diaries for 5 days. Both the groups, and particularly those with functional tremor, greatly overestimated how long their tremor was present, overestimating it by 65 and 28%, respectively, compared with actigraphy. 13 This indicates that symptom reporting can be markedly, and crucially *unintentionally* , inaccurate in functional disorders, with symptoms seeming to increase the more attention is directed toward them. Importantly it also illustrates that symptom reporting in non-functional or “organic” neurological disorders can also be inaccurate (over-reported), findings which are present in other neurological disorders such as epilepsy. 14 Clinicians and lawyers should therefore be cautious about using “credibility” of symptom severity as a proxy for willful exaggeration. People describe illness in very different ways, and their style of reporting is strongly shaped by the context and by what they think the assessment is for. It is commonplace for patients in routine clinical practice, as well as claimants in medicolegal settings, to omit, simplify, or amplify aspects of their story, even when they know that the assessor has access to their medical records. For example, a patient who is sitting relatively comfortably but rates his or her symptoms as “10 out of 10” in severity may be trying to communicate the overall impact of the symptoms, the worst level the patient has experienced in the past, or the sense of being overwhelmed by the situation, all of which is exacerbated by anxiety that his or her complaints will not be believed. These dynamics are particularly relevant in FND. People with FND frequently encounter skepticism, limited clinician knowledge, and patchy access to appropriate rehabilitation. Many report that the way their diagnosis is explained leaves them feeling that their symptoms are not real or not legitimate. 15 Against this background, it is understandable that some individuals respond by repeatedly seeking help and by accentuating the severity or impact of their symptoms to secure recognition, care, and validation. In other words, what may look like “exaggeration” can, at least in part, be a defensive or compensatory communication strategy in the face of perceived dismissal. 10

This is not to deny that a minority of patients with FND may also consciously feign or embellish symptoms. However, this is also the case for people with other neurological conditions, who may also exaggerate their symptoms. While there may some inaccuracy of symptom reporting, crucially individuals with FND will typically have a good idea of their level of disability, that is, what they can and cannot do or their intentional functioning. For example, they typically know if they can sometimes walk or stand and sometimes cannot. Occasionally, their movement might be better than they are aware of, but that should not impair their knowledge of how they can use their movement, for example, to stand or transfer or walk in the home or to the shop. Major inconsistencies between reported and observed function (e.g., a patient who reports an inability to walk but who is subsequently observed on video surveillance playing football or burlesque dancing) would therefore be in keeping with willful exaggeration or fabrication of symptoms and disability.

Definitively establishing that an individual is feigning the symptoms is possible only if the individual acknowledges willfully producing the symptoms, or if other evidence demonstrates a major inconsistency between reported and observed function. Detecting major inconsistencies therefore relies primarily on a thorough and well-prepared clinical interview, supported by a review of medical and forensic records, and ideally independent observations in real-life situations by other professionals involved in the treatment. Diagnosis is not based on a single encounter but requires triangulating evidence over time through interviews, third-party accounts, and longitudinal health records. Constructing a chronological summary of the patient's medical history is particularly valuable in assessing the credibility of reported symptoms and noting significant inconsistencies beyond the variability inherent in FND. It allows the assessment of reported complaints and clinic attendances that helps to clarify the relationship between an event and any subsequent symptoms attributed by the patient to the putative causal event. 11 Video surveillance and social media evidence, often supplied by insurers or legal teams, are commonly used to assess the credibility of a claimant's reported symptoms. When clear discrepancies arise between what a patient claims he or she cannot do and what the patient is observed doing, such evidence can significantly undermine the reliability of the patient's account. Another tool that is frequently used to aid the detection of inconsistencies is validity assessments, though these are not without problems of interpretation.

## Psychological Validity Assessments

Psychological validity assessments, such as Symptom Validity Tests (SVTs), Performance Validity Tests (PVTs), and effort tests, are widely used in the medicolegal evaluation of FND to help determine whether symptom presentation reflects intentional fabrication. 11 16 PVTs were originally developed to assess cognitive difficulties in individuals pursuing compensation following traumatic brain injury, with the aim of distinguishing genuine impairment from symptom exaggeration associated with malingering or factitious disorder. These tests have since been applied in broader contexts, including in the assessment of FND both inside and outside of litigation. PVT failure is typically defined by scoring below a set threshold, though this can be operationalized in various ways. One form of failure is performing below chance, suggesting deliberate selection of incorrect responses and possible evidence of intentional exaggeration. Alternatively, failure can be operationalized as abnormally poor scores on simple items, performance at chance level on complex items, or performance patterns inconsistent with mild-to-moderate neurological pathology. 17 In these contexts, poor performance might be interpreted as inadequate effort. However, the term “effort” has been criticized, as feigning poor performance may in fact require considerable cognitive exertion. 18 PVTs are now considered a standard part of neuro-psychometric evaluation, 19 and poor performance often casts doubt on the validity of the broader assessment. Yet, meta-analytic evidence shows that single-test PVT failures (particularly those *not* relying on the detection of performance below chance) are common across a range of neurological and psychiatric conditions including dementia, epilepsy, and moderate-to-severe TBI, and are not disproportionately elevated in individuals with FND. 20 This supports the view that reduced performance on validity testing is a general phenomenon, neither specific to FND nor necessarily consistent with fabrication or exaggeration of symptoms. Failures may instead reflect functional cognitive disturbances such as attentional difficulties, dissociative processes, behavioral problems such as impulsivity or heightened fatigue/pain/distress, rather than willful misrepresentation. These tools, although valuable, must therefore be used and interpreted with significant caution, and ideally within a broader evidentiary matrix rather than as standalone indicators of deliberate fabrication or exaggeration. However, it is worth noting that failure in these tests makes other cognitive tests difficult to interpret.

SVTs are often used alongside PVTs in the detection of malingering. 21 22 Some SVTs, like PVTs, rely on detecting improbable response patterns; others infer non-credibility when a claimant reports symptoms not typically associated with a given injury mechanism. However, the assumption that such symptom profiles indicate deception is contentious. Failure on SVTs does not inevitably signal feigning, particularly when symptoms are consistent with FND, which often follows triggering events like minor injuries or accidents, and by definition manifests with symptoms inconsistent with known disease pathologies or processes. Even positive signs used to diagnose FND, such as Hoover's sign or tremor entrainment, could in theory be misread as indicators of invalid performance if stripped of clinical context. Crucially, these signs do not by themselves distinguish between FND and feigning. Therefore, although psychological validity tests can provide valuable information in forensic evaluations, they must be interpreted cautiously. Poor performance on SVTs and PVTs should not be equated with deception unless corroborated by other evidence; rather, it may reflect the complex interplay of cognitive, psychological, and contextual factors that characterize FND.

## Causation

Once the disability caused by the FND is diagnosed and accepted as genuine, the question arises as to causation. In the UK law, causation is assessed using two key questions: “But for the defendant's actions or index event, would the injury or its severity have occurred?” and “Has the defendant's conduct or index event made a material contribution to the outcome?” Here “material contribution” means it made more than a minimal or trivial difference to the outcome (i.e., it need not be the sole or even principal cause, just a significant contributor). Sometimes an index event will be the sole cause, satisfying the “but for” test and resulting in full liability. When multiple factors contribute, the law distinguishes between divisible injuries, where harm increases with exposure and damages can be apportioned according to each contributor's share, and indivisible injuries, which are “all or nothing.” For indivisible injuries, any defendant or index event which made a material contribution may be liable for the whole injury, even if other causes are present. Recent case law confirms that the material contribution test may be applicable to both divisible and indivisible injuries, ensuring robust compensation where justified by expert evidence. This approach is directly relevant to conditions such as FND, where both the index event and other factors (psychological or physical) may interact, and careful medicolegal analysis is required to determine whether the defendant's actions crossed the threshold of material contribution. To address this question, one must address the extent to which predisposing, precipitating, and perpetuating factors contribute to the clinical presentation at the time of the medical examination ( Table 2 ). Ultimately however, determination of causation and apportionment rest with the court, and the legal tests applied can evolve with appellate authority over time.

**Table 2:** Causation and predisposing, precipitating, and perpetuating factors

Element	Description	Example/Notes
Predisposing factors (pre-injury)	Background vulnerabilities that increase the likelihood of developing FND after an injury (can be absent)	• Pre-existing functional or somatic disorders (e.g., chronic pain, irritable bowel–type symptoms, non-cardiac chest pain) • Other pre-existing disorders (e.g., migraine) • Neurodevelopmental conditions (e.g., autism spectrum disorder, attention deficit hyperactivity disorder) • Anxiety or depression • Stressful life events and trauma • Childhood adversity, bullying • Socioeconomic deprivation
Precipitating factors (at or around the injury)	Events that can trigger the onset of functional symptoms (e.g., resulting from accident or medical procedure) • Physical injury and/or • Psychological stress and/or • Events that change the behavior/attitudes of the subject or other people toward the subject (e.g., nocebo responses or maladaptive behaviors) The severity of physical injury is not linearly related to FND risk.	• Personal injury or other physical event that is experienced as painful/frightening/stressful. Example pairings include: - Limb injury/pain → functional weakness/complex regional pain syndrome-type picture - Faint or mild head injury +/− concussion → functional seizures - Mild head injury with concussion → functional cognitive disorder - Vestibular insult → functional dizziness (persistent postural perceptual dizziness) - Back injury/pain → functional weakness/gait disorder • Post-traumatic stress disorder • An event that reshapes behavior, expectations, and social responses in a way that can interact with vulnerability (e.g., prior anxiety, pain, dissociation) and tip the system into a functional disorder. Typical pairings include: - Mild head injury treated as moderate to severe head injury → functional cognitive disorder - Index event associated with poor medical care/feeling dismissed and injustice, humiliation, or loss of control - Index event leading to increased avoidant behavior, reduced activity, hypervigilance to bodily sensations, dissociative symptoms
Comorbidities	Conditions that frequently accompany FND and that may exacerbate overall presentation and disability.	• Depressive disorders • Anxiety and agoraphobia • Post-traumatic stress disorder • Sleep disturbance • Dissociative symptoms
Perpetuating factors—medical	Aspects of medical management that can maintain or worsen symptoms over time.	• Pain: Chronic pain and prolonged or escalating use of opioids and sedatives • Fatigue • Ongoing diagnostic uncertainty • Repeated or unnecessary investigations or procedures • Lack of clear explanation and coordinated rehabilitation • Abnormal illness beliefs or lack of acceptance of diagnosis of FND
Perpetuating factors—legal/contextual	Features of the medicolegal process and wider context that can drive chronicity.	• Ongoing litigation • Sense of injustice/anger • Repeated retelling of traumatic events • Perceived attacks on integrity (including surveillance) • Financial and social consequences of the claim • Reduced motivation to risk change/improvement while the case is unresolved.
Perpetuating factors—psychological/behavioral	Learned patterns that maintain FND beyond the initial trigger.	• Heightened inward monitoring of bodily sensations • Post-traumatic stress disorder • Activity avoidance because of fear of harm or symptom flare • Physical deconditioning • Reliance on aids and care • Reinforcement of a “sick role” • Loss of independence and role change

Abbreviation: FND, functional neurological disorder.

### Predisposing Factors

Current and previous aversive experiences (emotional neglect in childhood, sexual abuse, physical abuse, and stressful life events) contribute to a 4-fold increase in the odds of FND. However, about one-third of individuals with FND do not appear to have a history of aversive experiences, 23 and many in the general population also have a history of aversive experiences but do not develop FND. Previous illnesses, unexplained medical symptoms or functional somatic symptom disorder (e.g., chronic pain, irritable bowel syndrome, non-cardiac chest pain), anxiety, depression, or neurodevelopmental conditions (e.g., autism spectrum disorder) also increase the risk of developing FND in the future. Similarly, other factors such as bullying at work, stress at home, or social isolation may be relevant. However, these conditions are also quite common in the general population, and it is therefore also important to assess an individual's resilience in response to these conditions and previous injuries/crises. If the person's current symptoms and disability are thought to have been caused by the index event, this may be reinforced by an exploration of the person's history which shows that the person has been able to continue working and maintain relationships despite previous difficulties prior to the index event. Conversely, if prior to the index event they have developed significant worsening of symptoms like pain, fatigue, gastrointestinal issues, or mental health concerns, causing them to take extended time off work or breakdown in their relationships due to relatively minor injuries, events, or illnesses, this would be evidence against the index event being the sole cause.

### Precipitating Factors

Although FND can develop de novo without obvious precipitating factors, in 40 24 to 80% 24 25 of cases there may be a preceding physical injury. There is no linear relationship between the severity of the physical injury and the subsequent development of FND. Events associated with marked physical injury or significant psychological stress may lead to FND. On the other hand, if an individual suffers an extremely innocuous injury and the individual subsequently develops FND, it may be because of his or her profile of predisposing factors, suggested that the individual was going to develop FND anyway. Alternatively, in some individuals a seemingly mild injury can trigger FND in the presence or absence of predisposing factors. The nature and circumstances of the injury can also play a significant role in the development of FND. Here it is important to understand the broad mechanism through which FND is thought to arise. A compelling contemporary view is that functional neurological symptoms arise when the brain's normally accurate “predictive” machinery about movement and sensation goes wrong. 26 At one level, symptoms are thought to result from abnormally precise, involuntary prior expectations about movements or sensations, combined with excessive self-directed attention toward the affected body part. This combination can produce brain “processing errors” that are experienced as weakness, numbness, tingling, or abnormal movements. Physical events, including relatively minor injuries, can provide the critical sensory input that, when filtered through pre-existing vulnerabilities (such as past illness experiences, beliefs, and cognitive or affective biases), lays down an abnormal belief or expectation about movement or bodily integrity. If the injury is accompanied by marked panic or dissociation, the heightened arousal further increases the salience of bodily sensations, generates additional physical symptoms in its own right (for example, tremor), and powerfully conditions the link between attention, expectation, and symptom experience. From this perspective, FND can be understood as the expected, and largely involuntary, outcome of prior beliefs and focused attention acting on ambiguous bodily signals, rather than as a voluntary response, an idea that has important implications in medicolegal settings.

As a result of this mechanism there is some evidence that the nature of the injury may impact upon the type of FND that develops. For example, there is some evidence that functional paralysis or functional gait disorders is more common after back injuries/pain, 24 27 28 complex regional pain syndromes (a form of chronic pain) or functional dystonia in limbs affected by the injury, 27 29 and functional seizures or functional cognitive disorder after mild traumatic brain injuries and/or concussion. 25 30 In addition, based on this mechanism the way that others behave toward or treat an individual can precipitate the development of FND. Again, it is worth reiterating that in any of these cases FND does not result directly from structural damage to the nervous system.

Perceived poor medical care, injustice, humiliation, or loss of control adds a second hit. These experiences reinforce the sense that the body is unsafe and that others are not going to help. They may also promote rumination, repeated mental replay of the event, and bodily hypervigilance. This further strengthens the abnormal expectation (“this part of my body is damaged/not under my control”), so that, over time, the brain's predictions start to *drive* ongoing weakness, numbness, tremor, or other FND symptoms, even though the original physical insult was minor. Crucially, this process is automatic and involuntary, but it explains how an apparently trivial injury, in an emotionally charged and invalidating context, can precipitate FND in a vulnerable adult. There is clear evidence that invalidating and harmful medical interactions can significantly worsen and entrench FND, 31 and it is plausible, though less directly evidenced, that such experiences may also act as precipitants in vulnerable individuals. As an example, in our experience there are some cases of mild traumatic brain injuries that have been triaged and treated as moderate–severe traumatic brain injuries due to over-reporting of CT scans. The subsequent over-stated or confusing medical communication then shapes prior beliefs about serious head injury and focuses attention on the body, setting the stage for persistent FND-like (e.g., functional cognitive) symptoms in vulnerable adults.

A closely related concept, which may help in understanding the pathogenesis of FND following a minor injury, is that of “nocebo effect.” In nocebo, negative expectations/beliefs about a medical intervention led to negative outcomes. In simple terms, believing that something will or has injured or harmed oneself may be a self-fulfilling prophecy, in that this belief may materialize in very real symptoms. It may be useful to note that nocebo effect is the *opposite* of placebo effect, whereby positive expectations drive positive outcomes.

### Perpetuating Factors

FND can in some cases resolve quickly, but in other cases may persist and become chronic. The predisposing factors described earlier may also contribute as perpetuating factors. In addition, there are other factors that may contribute to the perpetuation of symptoms. Behavioral responses to injury can impact on recovery. Although rest in the immediate aftermath of injury may be important to recovery, excessive avoidance of activities for fear of worsening symptoms can lead to physical deconditioning, worsening of FND, and resulting anxiety and fatigue when a return to normal activities is attempted. This is not patient's fault and is better understood as a maladaptive behavior. In this context pain and fatigue are two of the most significant and interlinked factors, 32 but the approach to them should be nuanced. Although both can exacerbate FND symptoms and limit engagement with treatment, over-medication with painkillers can lead to paradoxically heightened sensitivity to pain in general, while over-activity or exertion can lead to “boom–bust” cycles of fatigue and pain. 33 Most treatment programs therefore emphasize a graded approach to re-engagement with normal activities. 33 34 Similarly, psychiatric illnesses such as anxiety disorders, depression, and post-traumatic stress disorder can both worsen the symptoms of FND, and again limit engagement with treatment. 32 The development of post-traumatic stress disorder (PTSD) after a traumatic event may not only predispose an individual to the development of FND, but it can also perpetuate FND symptoms. It has been proposed that PTSD and FND share overlapping mechanisms, including emotion dysregulation, altered interoception, heightened threat perception, and impaired fear extinction, which are likely to sustain symptoms in both conditions. 35 From a medicolegal perspective, the presence of PTSD after an index event strengthens the argument that event caused both psychological and physical symptoms.

Finally, the ongoing process of litigation can be a perpetuating factor that exacerbates symptoms. Legal processes, particularly those based in adversarial systems 36 (where legal disputes are framed as a contest between opposing parties and claimants often must prove suffering to receive compensation unlike inquisitorial or no-fault systems), can significantly prolong symptoms in individuals with FND following an initial triggering event. This is not merely a matter of financial incentivization; the litigation process itself can become a powerful context for symptom reinforcement. 12 Being repeatedly required to describe, justify, and re-experience one's symptoms, often in contested or skeptical settings, can heighten symptom salience and emotional distress. The adversarial nature of legal claims may also entrench the individual's identification with the sick role, such that symptoms may be unconsciously reinforced by internal needs (e.g., a desire for justice or validation) and/or by external cues (e.g., advice from legal representatives or expectations from family). Additionally, ongoing medical assessments and expert evaluations, though intended to clarify, may introduce iatrogenic harm by increasing uncertainty or invalidating symptoms. In this way, the legal context functions not so much as a motivator but more as a system that maintains and amplifies symptoms through repeated focus, emotional strain, and social positioning. It is however important to note that these dynamics do not solely apply to disorders like FND but equally apply in cases of *organic* or physically verifiable disorders. Moreover, studies that have evaluated symptom burden after the cessation of litigation process in personal injury cases have shown that many individuals remain symptomatic from symptoms such as chronic headache or others that lack a clear structural basis but are associated with high levels of subjective distress and disability. 37 38 The persistence of symptoms unrelated to structural damage following cessation of litigation undermines the presumption of malingering for financial gain and rather highlights the authenticity and complexity of this group of conditions.

## Treatment and Prognosis

The treatment approach to FND should be individualized and approached in a hierarchical way. 39 It begins with a clear and confident explanation of the diagnosis by a neurologist or neuropsychiatrist, ideally accompanied by demonstration of positive clinical signs and supplemented by accessible patient resources. While some patients may improve with explanation alone, others, particularly those with more severe or persistent symptoms, require tailored interventions like physiotherapy or cognitive behavioral therapy (CBT), both of which have evidence of effectiveness. It is essential that these therapies are delivered by clinicians experienced in FND, as treatment approaches differ significantly from those for structural or musculoskeletal disorders or other psychological disorders such as anxiety. Physiotherapy for FND focuses on restoring automatic movement and reducing maladaptive attentional focus, while CBT targets unhelpful thought patterns and avoidance behaviors that perpetuate symptoms. Ultimately, both approaches aim to reduce fear and re-engage patients with normal functioning. Problematic FND causing persistent or recurrent symptoms and requiring specialist treatment rarely happens in isolation. In most cases, patient's management is best approached in a multidisciplinary way, involving neurologists, psychiatrists, and allied health care professionals such as physiotherapists, psychologist, and/or occupational therapists, in accordance with individual needs. Treatment may be delivered in an outpatient, inpatient, or virtual setting (or in some combination of these), again depending on individual variables. Sometimes co-existing problems such as post-traumatic stress disorder, migrainous headaches, or chronic pain may need to be treated first, before undergoing specific treatment for FND.

Functional neurological disorders are potentially reversible, but outcomes can be variable. The prognosis for FND severe enough to be referred to a neurology clinic is generally regarded as unfavorable, particularly in tertiary care settings where chronic, disabling symptoms are frequently encountered. 40 41 42 However, it is not clear how applicable the findings of these studies are to individuals presenting with FND in the context of litigation. For untreated functional motor symptoms (FMS) such as functional limb weakness, the prognosis is often poor, with symptoms remaining the same or worsening in the majority of patients, 42 and a systematic review reporting a remission rate of only 20%. 41 42 This persistence is confirmed by long-term follow-up data on functional limb weakness, showing that 80% of patients still had weakness after an average of 14 years. 40 Similarly, the overall prognosis for functional seizures is generally poor, with the remission rate in pooled studies being approximately 33%. 42 However, defining the true “untreated” natural history is challenging because many patients receive treatment even in studies classified as examining natural course. Regarding specific treatments, a large randomized controlled trial (Physio4FMD) comparing specialist physiotherapy with treatment as usual for functional motor disorder found no significant difference between groups for improved physical functioning at 12 months. Nevertheless, the specialized physiotherapy group was twice as likely to self-report an improvement in their motor symptoms compared with the control group (59% versus 38%), and reported better scores on measures of mental health and confidence in the diagnosis. 43 For functional seizures, specific CBT plus standardized medical care did not result in a statistically significant reduction in monthly seizure frequency compared with standardized medical care alone in a major randomized trial (CODES). 44 However, the CBT group showed significant improvements in critical secondary outcomes, including a longer period of seizure freedom, better self-rated health-related quality of life, less impairment in psychosocial functioning, and greater self-rated and clinician-rated global improvement. Frequently reported good prognostic factors include short duration of symptoms prior to diagnosis, early diagnosis, and young age. Furthermore, for functional motor disorder, patients receiving specialist physiotherapy, a greater perception of having control over recovery predicted improvement, 45 while for functional seizure patients, CBT was more effective in reducing seizure frequency among those with a higher number of somatic symptoms or at least one current comorbid psychiatric diagnosis. 46 The latter finding demonstrates a key nuance, which is although high comorbidity often predicts a worse natural course, it may indicate a subgroup that benefits most from complex, tailored interventions like CBT. Crucially, the idea that many prognostic factors are weak or inconsistent is also widely supported. Many commonly reported prognostic factors, such as symptom duration, pain, fatigue, being in receipt of benefits, and educational level, were not consistently found to influence treatment outcome in the Physio4FMD trial of specialist physiotherapy for FMD. This highlights the ongoing challenge in predicting outcome, which is frequently an important question in medicolegal reports. This difficulty in predicting outcomes is underscored by the aforementioned studies which demonstrate that a large proportion of the variance in outcomes remains unexplained. This unexplained variance emphasizes that FND is a highly heterogeneous condition and that prognosis and treatment planning need to be individualized rather than derived mechanistically from group averages. With this in mind the expert may need to provide the court with a range of possible outcomes and different care needs.

The litigation process itself can create a difficult context for rehabilitation. Claimants are repeatedly required to describe their injury, symptoms, and disability, and may perceive a financial incentive to emphasize ongoing problems, which can sit uneasily alongside the task of focusing on recovery. While individuals may superficially engage with treatment during litigation, meaningful, sustained improvement may be uncommon, even though there are exceptions. This has implications for how risk is shared between claimant and defender. If treatment occurs before settlement and proves only partially effective or unsuccessful, the defender may be required to settle based on long-term disability despite having funded therapy. Conversely, if settlement is reached on the assumption that treatment will be beneficial and rehabilitation is only sought afterwards, the claimant effectively carries the risk that improvement may be less than anticipated. For some individuals it is therefore more pragmatic to defer major rehabilitation efforts until after conclusion of proceedings, when there is greater financial security and less perceived conflict between the goals of litigation and the goals of treatment. However, where appropriate, earlier treatment may be appropriate and provide useful information about prognosis.

## Ongoing Care and Aids

The question of whether ongoing care is needed in FND often arises during the process of a legal claim. In line with occupational therapy consensus recommendations, 34 this should be assessed on an individual basis, recognizing a potential tension between necessary support and the risk that excessive or overly passive care, including indiscriminate provision of aids and adaptations, can impede rehabilitation and reinforce disability in some individuals. Ideally, the goals of care should be explicitly rehabilitative, supporting the person to regain independence with a graded reduction in assistance over time. In practice, care arrangements are not always organized in this way, but for those with severe and persistent FND, or those who have not benefited from appropriate treatment, provision of care and disability adaptations should be broadly similar to that offered for other neurological conditions, with the acknowledgment that improvement may still be possible and periodic review of care needs remains appropriate.

## Conclusion

Functional neurological disorder presents particular challenges in medicolegal practice, but these challenges are manageable with a careful, evidence-based approach. Positive clinical signs and contemporary neurobiological models support FND as a legitimate diagnosis, yet they do not exclude the possibility of exaggeration, factitious disorder, or malingering in individual cases. Symptom validity and performance validity tests can provide useful corroborative information, but they are neither necessary nor sufficient to infer intentional deception and must be interpreted in the context of the whole clinical picture. Likewise, litigation, compensation, and other external incentives may contribute to the onset or persistence of symptoms in some individuals, but they are rarely the sole explanation and should be weighed alongside pre-existing vulnerabilities and the nature of the precipitating event. The task of the expert is therefore not to advocate for a particular narrative, but to offer a transparent, balanced opinion on diagnosis, causation, and prognosis, clearly separating areas of consensus from areas of uncertainty. Done well, this approach respects the duties owed to the court while minimizing the risk of both unwarranted skepticism and uncritical acceptance of claimed disability.
